# Mental health in individuals with self-reported psychiatric symptoms during the COVID-19 pandemic: Baseline data from a swedish longitudinal cohort study

**DOI:** 10.3389/fpsyt.2022.933858

**Published:** 2022-07-19

**Authors:** Alexander Rozental, Karolina Sörman, Olivia Ojala, Simon Jangard, Samir El Alaoui, Kristoffer N. T. Månsson, Shervin Shahnavaz, Johan Lundin, David Forsström, Maria Hedman-Lagerlöf, Tobias Lundgren, Nitya Jayaram-Lindström

**Affiliations:** ^1^Department of Clinical Neuroscience, Centre for Psychiatry Research, Karolinska Institutet, Stockholm Health Care Services, Region Stockholm, Solna, Sweden; ^2^Department of Psychology, Uppsala University, Uppsala, Sweden; ^3^Great Ormond Street Hospital Institute of Child Health, University College London, London, United Kingdom; ^4^Division of Psychology, Department of Clinical Neuroscience, Karolinska Institutet, Solna, Sweden; ^5^Department of Psychology, Stockholm University, Stockholm, Sweden

**Keywords:** COVID-19, mental health, psychological impact, anxiety, depression, isolation

## Abstract

**Objective:**

Individuals with psychiatric disorders may be both vulnerable and sensitive to rapid societal changes that have occurred during the COVID-19 pandemic. To fully understand these impacts, repeated measurements of these individuals are warranted. The current longitudinal study set out to perform monthly assessment of individuals with common psychiatric disorders using established questionnaires with a possibility for them to self- rate their symptoms, over time.

**Methods:**

Recruitment of individuals who identified themselves as struggling with mental health problems, living in Sweden between July 2020 and June 2021 using an online survey. The individuals answered questions on demographics, psychiatric history, current psychiatric symptoms (e.g., Patient Health Questionnaire, PHQ-9; General Anxiety Disorder, GAD-7), somatic health, health-care contacts and any changes therein during the pandemic. Monthly, longitudinal assessments are still ongoing (consenting participants provide data for 1 year), and here we present descriptive statistics from the baseline measurement. All measurements from baseline (>400 items), and follow-ups are presented in detail.

**Results:**

A total of 6.095 participants (average age 35 years) submitted complete baseline data. Marital status (43% single) and number of years of education (48% highest degree being high school) were evenly distributed in this population. The most common lifetime psychiatric disorder in the sample was depressive disorder (80.5%) and generalized anxiety disorder (45.9%), with a substantial proportion having severe symptoms of depression. (30.5%) and anxiety (37.1%). Lifetime suicidal ideation (75.0%) and non-suicidal self-harm (57.7%) were prevalent in the group and 14.5% reported drug use during the pandemic. Allergies (36.8%) were the most common somatic condition, followed by irritable bowel syndrome (18.7%). For those having experienced a traumatic event, 39% showed symptoms during the pandemic indicating PTSD. Regarding contact with mental health services during the pandemic, 22% had established a new contact, and 20% reported to have increased their psychiatric medication compared to before the pandemic.

**Conclusion:**

Baseline data collected during the pandemic from individuals in Sweden with pre-existing psychiatric disorders demonstrate that this sample represents a population suitable for an investigation on the long-term impact of the pandemic, as intended by the longitudinal investigation that is ongoing. Follow-up questionnaires over a 12-month period are being collected and will indicate how the health and well-being of this population was impacted during the changes and uncertainties that have been characteristic of the past 2 years.

## Introduction

The COVID-19 pandemic has brought about significant changes in our society – some potentially more long-lasting than others. Not only has the SARS-CoV-2 virus resulted in a significant number of fatalities, but also led to the development of a range of heterogeneous psychological and somatic symptoms, with possible long-term consequences not yet fully understood.

In Sweden the statistics related to the COVID-19 pandemic, indicate close to 2.5 million confirmed infections, approximately 9200 cases requiring intensive care, and more than 18,500 casualties (1). Apart from having an impact on the psychological well-being of the general population, the pandemic is believed to have been particularly adverse for individuals with ongoing or previous mental health problems (2–5). In order to fully understand how mental health has been affected among vulnerable groups, experts in the field expressed an urgent need for developing principles of good practice in researching the pandemic (6). These include publishing study protocols, sharing information on study measures, and rapid and real-time dissemination of results, with the purpose of allowing comparisons between samples, populations, and countries.

In an early study in China, Hao et al. (7) evaluated the effect of immediate stress on persons with and without psychiatric patients during the peak of the pandemic and in conjunction with a strict period of lockdown. Not surprisingly the psychiatric patients demonstrated an increase in anxiety, depression, stress levels, and more than one-third also fulfilled the criteria for post-traumatic stress disorder (PTSD). The psychiatric population was also more significantly worried about their physical health. Given that many individuals with psychiatric disorders live alone, they could also be more susceptible to feelings of isolation, furthering the deterioration of their psychiatric symptoms (8). In particular, feelings of loneliness and isolation could worsen psychiatric symptoms and even increase the risk of self-harm and suicide if not detected at an early stage (6). The study by Hao et al. (7) was among the first to highlight that individuals with psychiatric problems are a vulnerable population in the context of the pandemic and resulting lock downs, where adaptations need to be made within the health care services to better meet their needs (e.g., telepsychiatry, home delivery of medication, online first-aid resources to support infection management).

In a Swedish study within psychiatric services, Flygare et al. (9) telephoned 1071 psychiatric patients registered in the clinic, who had not been in contact with their outpatient care facility during the early phases of the pandemic. Most patients (81%) reported that they did not experience a deterioration in psychological well-being, and of those who did (19%), psychiatric management plans were already put into place and deemed sufficient by the respondents. Titov et al. (10) assessed the psychiatric symptoms of those seeking outpatient care at a digital outpatient mental health service in Australia pre (*n* = 1650) and during (*n* = 1668) the outbreak of the COVID-19 pandemic and reported a small increase in anxiety severity, as well as an increased number of individuals reporting a recent onset of anxiety and depression. The access and frequency of interaction with healthcare providers, is an important factor that could have impacted the health and well-being for individuals with psychiatric disorders. It is common that individuals with chronic psychiatric symptoms require renewed prescription and regular support in managing their symptoms, as well as help concerning diet and lifestyle factors. During the pandemic, some of these services may have been more difficult to access during the lock downs and during more severe periods of infection spread, warranting further studies within and between countries to inform the development of future health care structures. Taken together, these studies indicate that in the face of stressful events such as the pandemic, individuals with current or previous episode(s) of psychiatric disorders may experience an increase in their symptom load, requiring specific attention and possibly different types of structures (e.g., online and combined) to address their care needs.

Pre-existing psychiatric disorders could also increase the risk to get infected by viral infections or make the outcomes worse (11–15). One possible biological explanation for this is that COVID-19 disease represents a multiorgan pathology, affecting the central nervous system and leading to neuroinflammation. While it is still unclear if the virus itself exacerbates existing psychiatric symptoms, the sensitive interplay between the viral-induced neuroinflammation in the central nervous system resulting from COVID-19, and underlying neuroinflammation related to existing psychiatric symptoms, may be a possible explanation for the aggravated effect in this sensitive population (16). Other possible explanations as to why psychiatric patients show an elevated risk in getting infected could be a low risk- awareness, difficulties in complying with preventive behaviors (e.g., wearing masks and maintaining the needed hygiene standards), and unstable housing situations. Heightened stress and anxiety due to rapid societal changes related to the pandemic and intense media information load could also further exacerbate existing psychiatric symptoms (17).

However, the consequences of the pandemic among individuals with psychiatric disorders remain unclear. The research mentioned above, points toward a worsening of symptoms and psychological well-being, but it should be noted that some studies have not been able to detect this trend [see (18)]. Most investigations have so far been cross-sectional by design, and lack recurrent and long-term follow-ups, making it difficult to determine if the well-being of individuals with psychiatric disorders have worsened over the course of the pandemic. Furthermore, it is not known if the psychological well-being in this group of individuals might also have changed due to other external factors, such as societal restrictions and employment losses. Longitudinal studies with more comprehensive self-assessments are therefore warranted, allowing a more reliable and valid investigation of how pandemic related regulations, and change in care structures, have affected this vulnerable group in society.

In order to tackle some limitations in the current literature, we performed a nation-wide longitudinal data collection in Sweden. The intention is to present researchers, clinicians, and decision-makers with a database from which future research on the effects of the pandemic in individuals struggling with psychiatric disorders, can extend from. The current study focuses on the comprehensive baseline demographics of individuals with pre-existing psychological symptoms who participated in the study, describing their symptom profile in relation to the pandemic, their ability to access mental health services, any changes in their existing care, and general aspects such as the impact on their families and ability to stay connected with the larger community.

## Materials and methods

### Study design

This project at the Karolinska Institutet constitutes a collaboration with a United Kingdom research initiative called the Repeated Assessment of Mental Health in Pandemics (RAMP). This collaboration creates an opportunity for comparisons of data between countries, its population and possible differential impact due to country specific and regulatory specific differences and with the advantage of utilizing similar scales and questionnaires. The study has therefore included a majority of the questionnaires in the RAMP study and several specific scales of interest to the Swedish research team (all details provided below). For more details on the RAMP, see https://www.kcl.ac.uk/research/ramp. The current paper focuses on comprehensive baseline data collected in Sweden between July 2020 and June 2021, in individuals from the community, with current or previous episode(s) of psychiatric disorders. Later on, the results from the longitudinal investigation will be able to explore how this population responded during the pandemic, which in turn may help to identify risk and specific care needs for this particular group.

### Participants

In this study, 6095 individuals are included from the community. Eligibility criteria were: (1) being 18 years or older, (2) living in Sweden, and (3) having a current or lifetime experience of psychiatric symptoms. Given the nature and timing of the study, significant efforts were placed on the recruitment process and in creating awareness for the study. This was done in several ways. First, to recruit a representative sample for this study from the general population, we actively worked with Non-Governmental Organizations working with mental health and with psychiatric clinics in the country, to spread information about the study. Second, we used Facebook and Instagram as portals to showcase a variety of advertisements which actively catered to diverse populations, gender, and different age groups. Public awareness of the project was made possible through presentations in the Swedish news (i.e., television and radio) as well as public presentations at a meeting held by a patient caregiver association, the Swedish Partnership for Mental Health. These measures were considered essential to increase outreach and engagement from this specific population, during the ongoing pandemic to participate in an online survey study.

## Procedures

For a complete overview of the study structure, the scales and measures utilized in the full study, see Table 1. An online survey was made publicly available through a study website,^1^ collecting data on psychiatric symptoms, changes in access or need for mental health services as well as the overall impact on general health and well-being in relation to the pandemic for individuals who identified themselves as struggling with psychiatric disorders. To assess pre-existing or life time diagnosis, participants were asked to select from a list of psychiatric diagnosis and had the possibility of selecting all the categories that applied. This type of design is similar to other international studies conducted during the pandemic (19, 20). The study data was collected and managed via Research Electronic Data Capture (REDCap), an electronic data tool, hosted at the Karolinska Institutet (21, 22).

**TABLE 1:** Overview of data collection structure.

	Baseline and follow-up in months												
Measures	B	1	2	3	4	5	6	7	8	9	10	11	12
Patient Health Questionnaire-9 Items (PHQ-9)	●	●	●	●	●	●	●	●	●	●	●	●	●
Generalized Anxiety Disorder-7 Items (GAD-7)	●	●	●	●	●	●	●	●	●	●	●	●	●
Clinical Outcomes in Routine Evaluation (CORE-10)	▶	▶	▶	▶	▶	▶	▶	▶	▶	▶	▶	▶	▶
The Alcohol Use Disorders Identification Test (AUDIT)	●	■		■		■		■		■		■	
The Drug Use Disorders Identification Test (DUDIT)	●		■		■		■		■		■		■
The Posttraumatic Stress Disorder Checklist PCL-5	●						●						●
The Barkley Adult ADHD Rating Scale-IV (BAARS-IV)	▶												
Short Health Anxiety Inventory (SHAI)-14	▶												
The Community Assessment of Psychic Experiences-Positive Scale (CAPE-5)	▶												
Panic disorder (DSM-5 Panic)	●												
Obsessive-Compulsive Inventory-Revised (OCI-R)	●												
UCLA Loneliness Scale	▶						▶						▶
Resilience behaviors during COVID-19 (Brief Resilience Scale)	●						●						●
Prolonged Grief Disorder (PG-13)	■						●						●
Self-care behaviors during COVID-19	●						●						●
Pandemic-related worries	●						●						●
Pure Procrastination Scale (PPS)	●						●						●
Problem Gambling Severity Index (PGSI)	●												●
Self-harm	▶												
Prosocial Tendencies Measure (PTM)	●												
Demographic, social, and health care items	●	▶	▶	▶	▶	▶	▶	▶	▶	▶	▶	▶	

● Full instrument; ■ screening questions; ▶ selected items.

B, baseline. The remaining measures not reported in the main article include Brief Resilience Scale (24); Clinical Outcomes in Routine Evaluation (50), three selected items (2, 3, and 4: “Felt I have someone to turn to for support when needed,” “Felt able to cope when things go wrong,” and “Felt talking to people is too much for me”); UCLA Loneliness Scale, four items used, as in 51; PPS (52); panic disorder (53); Short Health Anxiety Inventory (54), selected the three highest loading items on each of the three factors (illness likelihood, illness severity, body vigilance = 9 total items), the study does not have items 1, 4, 13, and 14 from original SHAI, but have added four questions from HAI; Obsessive-Compulsive Inventory-Revised (55); Problem Gambling Severity Index (56), asked about past month instead of year and added two screening questions; The Community Assessment of Psychic Experiences-Positive Scale (57; 58), items 31, 33, 34, 41, and 42 from the CAPE; Prolonged Grief Disorder (Prigerson and Maciejewski); Short version of The Barkley Adult ADHD Rating Scale-IV (60); Self-harm (36); and Prosocial Tendencies Measure (62).

After consenting to the study, participants were given the option to also complete monthly follow-up questionnaires over a 12-month period (by stating their email address). Personal integrity of the participants was maintained by allocating a unique study identification number to each participant upon entry to the study.

### Measurements

The study included a range of measures on psychiatric symptoms, somatic health, and general well-being. In the current study we present an overview of selected measures (Table 1) on the most prevalent and major psychiatric symptoms in this population. Completion of the baseline battery of questionnaires took 30–50 min. The REDCap structure was set up such that the questionnaires had to be filled out sequentially and items were compulsory to fill out before moving on the next questionnaire. A present card worth SEK 150 was provided to those participants who completed the longitudinal data collection. To understand the severity of psychiatric symptoms in the study population, we have added information about healthy norm data for the original scales from previous studies, with data collected before the pandemic (presented in Tables 2, 3).

**TABLE 2:** Psychiatric symptom load during COVID-19.

	Total (*n* = 4513^a^)	Female (*n* = 3210)	Male (*n* = 1072)	Non-binary (*n* = 231)	Norm data
*Alcohol Use Disorder (AUDIT)*	*n (%)*	*n (%)*	*n (%)*	*n (%)* ^b^	*%* Female/Male
None	3891 (86.2)	2802 (87.3)	887 (82.7)	202 (87.5)	88.2/79.1
Risky alcohol use	529 (11.7)	351 (10.9)	152 (14.2)	26 (11.3)	10.8/17.9
Hazardous alcohol use	57 (1.3)	36 (1.1)	20 (1.9)	1 (0.4)	NA/NA
Alcohol dependence	36 (0.8)	21 (0.7)	13 (1.2)	2 (0.9)	1.0/3.0
	**Total (*n* = 4508^a^)**	**Female (*n* = 3206)**	**Male (*n* = 1071)**	**Non-binary (*n* = 231)**	**Norm data**
*Substance Use Disorder (DUDIT)*	*n (%)*	*n (%)*	*n (%)*	*n (%)* ^b^	*%* Female/Male
None	3855 (85.5)	2773 (86.5)	895 (83.6)	187 (81.0)	98.4/95.2***
Drug related problems	611 (13.6)	412 (12.9)	158 (14.8)	41 (17.8)	1.6/4.8***
Drug dependence	42 (0.9)	21 (0.7)	18 (1.7)	3 (1.3)	NA/NA

AUDIT, Alcohol Use Disorder Identification Test; DUDIT, Drug Use Disorder Identification Test.

^a^Corresponding to 74% of all participants.

^b^We used the lower cutoff values from the female category for individuals identified as non-binary.

**TABLE 3:** Psychiatric symptom load during COVID-19.

	Total (*n* = 4702*^a^*)	Female (*n* = 3346)	Male (*n* = 1117)	Non-binary (*n* = 239)	Norm data
*Anxiety disorder (GAD-7)*	*n (%)*	*n (%)*	*n (%)*	*n (%)*	*%* Female/Male
No anxiety	513 (10.9)	322 (9.6)	165 (14.8)	26 (10.9)	67.1/74.4
Mild anxiety	1185 (25.2)	819 (24.5)	310 (27.8)	56 (23.4)	26.1/20.2
Moderate anxiety	1259 (26.8)	913 (27.3)	284 (25.4)	62 (25.9)	5.3/4.4
Severe anxiety	1745 (37.1)	1292 (38.6)	358 (32.1)	95 (39.8)	1.3/0.7
	**Total (*n* = 4984*^b^*)**	**Female (*n* = 3568)**	**Male (*n* = 1167)**	**Non-binary (*n* = 249)**	**Norm data**
*Major depressive disorder (PHQ-9)*	*n (%)*	*n (%)*	*n (%)*	*n (%)*	*%* Female/Male
Minimal or no depression	297 (6.0)	198 (5.6)	88 (7.5)	11 (4.4)	87.1/91.7
Mild depression	812 (16.3)	594 (16.7)	187 (16.0)	31 (12.5)	NA/NA
Moderate depression	1108 (22.2)	794 (22.3)	256 (21.9)	58 (23.3)	12.9/8.3
Moderately severe depression	1246 (25.0)	889 (24.9)	280 (24.0)	77 (30.9)	NA/NA
Severe depression	1521 (30.5)	1093 (30.6)	356 (30.5)	72 (28.9)	NA/NA
	**Total (*n* = 3159)**	**Female (*n* = 2284)**	**Male (*n* = 699)**	**Non-binary (*n* = 176)**	**Norm data**
*Posttraumatic Stress Disorder (PCL-5)*^c^**	*n (%)*	*n (%)*	*n (%)*	*n (%)*	*%^d^*
Minimal or no symptoms	1902 (60.2)	1360 (59.5)	443 (63.4)	99 (56.3)	89.7
Posttraumatic stress disorder	1257 (39.8)	924 (40.5)	256 (36.6)	77 (43.8)	10.3

GAD-7, Generalized Anxiety Disorder-7; PHQ-9, Patient Health Questionnaire-9; GAD-7, Generalized Anxiety Disorder-7; PCL-5, Posttraumatic Stress Disorder Checklist-5.

^a^Corresponding to 77% of all participants.

^b^Corresponding to 82% of all participants.

^c^Only participants screening for a traumatic experience answered these questions.

^d^Gender neutral norms.

#### Demographic variables

Demographic information at baseline included gender, age, country of birth, occupation, life-time diagnosis, education, health-care contacts, social life, and perceived isolation.

#### Lifestyle behaviors and resilience

Pandemic related lifestyle behaviors were measured through a novel questionnaire developed by RAMP (23). The questionnaire entails 14 items out of which 7 items are presented in this study: Hygiene; Sleep; Leisure activities; Social activities; Alcohol; Cannabis; and Smoking. The items were assessed for the past 2 weeks and rated on a 5-point scale, corresponding to “not at all” (0), “one or two days” (1), “several days” (2), “more than half the days” (3), or “nearly every day” (4).

Resilience was measured through the Brief Resilience Scale [BRS; (24)]. The questionnaire entails six items concerning the perceived ability to recover from stress. BRS is rated on a 5-point scale answering statements and corresponding as “strongly disagree” (1), “disagree” (2), “neutral” (3), “agree” (4) or “strongly agree” (5). The total score is calculated by reverse coding items 2, 4, and 6, then summarizing the total score and calculating the mean of the six items. The BRS has shown good internal consistency, α = 0.80–91 (depending on sample), and test-retest reliability, Intraclass Correlation Coefficient of 0.69 (24).

#### Anxiety disorder

Symptoms of anxiety were measured with the Generalized Anxiety Disorder-7 Items [GAD-7; (25)]. The GAD-7 is a validated questionnaire which measures symptoms of anxiety during the past 2 weeks with seven self-rating items on a 4-point scale, from 0 to 3. Scoring of symptoms were categorized as None (female/male/non-binary: 0–4 points); Mild anxiety (females/males/non-binary: 5–9 points); Moderate anxiety (females/males/non-binary: 10–14 points); Severe anxiety (females/males/non-binary: 15–21 points) in accordance with Spitzer et al. (25). Healthy norm data were derived from Löwe et al. (26). In the current study, internal consistency was Cronbach’s α = 0.90.

#### Major depressive disorder

Depressive disorder was measured using the Patient Health Questionnaire-9 [PHQ-9; (27)] consisting of nine items assessing symptoms for the past 2 weeks. Scoring of symptoms were categorized as Minimal or no depression (female/male/non-binary: 0–4 points); Mild depression (females/males/non-binary: 5–9 points); Moderate depression (females/males/non-binary: 10–14 points); Moderately severe depression (females/males/non-binary: 15–19 points); and Severe depression (females/males/non-binary: 20–27 points) in accordance with Kroenke et al. (27). PHQ-9 has shown satisfactory psychometric properties and adequate validity as a measure of depression (27, 28). In the current study, internal consistency was α = 0.88. Healthy norm data were derived from Johansson et al. (29).

#### Posttraumatic Stress Disorder

Symptoms of posttraumatic stress were assessed using the Posttraumatic Stress Disorder Checklist PCL-5 (30). The questionnaire comprises 20 items corresponding to the DSM-5 PTSD symptom criteria. Participants rated the intensity of each symptom during the past month using a 5-point scale from 0 (“not at all”) to 4 (“extremely”). Evaluations of the PCL-5 indicate adequate test-retest reliability and validity (31). A Swedish version has demonstrated satisfactory psychometric properties (32). Scoring was categorized as Minimal or no symptoms (females/males/non-binary: 0–37 points); and PTSD (females/males/non-binary: 38–80 points) in accordance with Weathers et al. (30). Healthy norm data were derived from Blevins et al. (31). In the current study, internal consistency was α = 0.95.

#### Alcohol use disorder

Symptoms of alcohol use disorder were assessed using the Alcohol Use Disorders Identification Test [AUDIT; (33)]. AUDIT consists of 10 items where the first eight questions assess consumption and the last two alcohol-related harms. The first eight questions are measured on a 5-point scale ranging from 0 to 4, whereas the last two questions are measured on a 3-point scale rated as 0, 2, or 4. The total score is calculated by summarizing the ratings and could range between 0 and 40. Scoring of symptoms were categorized as None (female: 0–5 points; male: 0–7; non-binary: 0–5); Risky alcohol use (female: 6–13 points; male 8–15 points; non-binary: 6–13 points); Hazardous alcohol use (female: 14–17 points; male 16–19 points; non-binary: 14–17 points); and Alcohol dependence (female: 18–40 points; male: 20–40 points; non-binary: 18–40 points), in accordance with Berman et al. (33). Healthy norm data were derived from Bergman and Källmén (34). In the current study, internal consistency was α = 0.77.

#### Substance use disorder

Symptoms of substance use disorder were measured using the Drug Use Disorders Identification Test [DUDIT; (33)]. DUDIT contains 11 items and the first nine questions concern consumption, rated on a 5-point scale ranging from 0 to 4, and the last two items concern drug-related harms, rated on a 3-point scale scored as 0, 2, or 4. The total score is calculated by summarizing the ratings and could range between 0 and 44. Scoring of symptoms were categorized as None (female: 0–1 points; male: 0–5; non-binary: 0–1); Drug related problems (female: 2–24 points; male: 6–24; non-binary: 2–24); and Drug dependence (female/male/non-binary: 25–44 points) in accordance with Berman et al. (33). Healthy norm data were derived from Berman et al. (35). In the current study, internal consistency was α = 0.95.

#### Self-harm

Self-harm was assessed based on the questions from the Thoughts and Feelings questionnaire (36), revised in this study through separating self-harm with or without suicidal intention and asking specifically about suicide ideation. The presence of life-time suicide ideation, non-suicidal self-harm, and suicide attempts were measured through answering “yes” or “no” to the following questions: “Have you ever thought about ending your life?” (suicidal ideation), “Have you ever deliberately harmed yourself (e.g., though cutting, biting or hitting yourself) without the intention to end your life?” (non-suicidal self-harm), and “Have you ever deliberately harmed yourself (e.g., through cutting, biting, or hitting yourself or taking pills) with the intention to end your life?” (suicide attempt).

### Statistical analysis

Descriptive statistical analyses and figures were performed using Stata/MP v15.1 (2020; StataCorp, College Station, TX, United States). All the Stata commands and results are available at www.github.com/neuronsson/CPF_COVID19_ 2022/to provide a complete and detailed output.

### Ethical consideration

This study was conducted in accordance with the Declaration of Helsinki and approved by the Swedish Ethical Review Authority (Dnr: 2020-02798). All participants provided electronic informed consent before participation and completing the questionnaires.

## Results

### Demographics and clinical characteristics

Table 4 presents the demographics of the study population. A total of 93.3% reported that they were born in Sweden. Of all participants, 93.3% also reported that they had at least one life-time psychiatric disorder, and the average number of diagnoses per individual was 3.13 (SD = 2.07). The proportion of participants meeting criteria for one, two, and three life-time psychiatric disorders were 17.6, 22.9, and 21.3%, respectively. The most common psychiatric disorders reported were major depressive disorders (80.5%), generalized anxiety disorder, (45.9%), and panic disorder (42.1%). More detailed information of life-time psychiatric disorders and suicidal behaviors are presented in Table 5. Regarding concomitant general health issues, only 2343 (38.4%) of the participants reported that they did not suffer from chronic somatic conditions (e.g., irritable bowel syndrome and diabetes). The most common somatic conditions were allergies (36.8%), irritable bowel syndrome (18.7%), and lung or breathing problems (16.0%). In addition, 0.9% had diabetes type 1, and 3.0% had diabetes type 2.

**TABLE 4:** Demographics of study population.

	Total (*n* = 6095)	Female (*n* = 4304)	Male (*n* = 1490)	Non-binary (*n* = 301)
Age, *M* (SD)	35.05 (12.1)	34.54 (12.1)	37.45 (12.2)	30.4 (9.8)
Age groups, *n* (*%*)				
18–27	1934 (31.7)	1458 (33.9)	342 (23.0)	134 (44.5)
28–37	1944 (31.9)	1345 (31.3)	489 (32.8)	110 (36.5)
38–47	1892 (31.0)	1278 (29.7)	561 (37.7)	53 (17.6)
48+	325 (5.3)	223 (5.2)	98 (6.6)	4 (1.3)
Education (highest level), *n* (*%*)				
Elementary school	457 (7.5)	297 (6.9)	126 (8.5)	34 (11.3)
High school	2457 (40.3)	1693 (39.3)	648 (43.5)	116 (38.5)
University	3181 (52.2)	2314 (53.8)	716 (48.1)	151 (50.2)
Employment status before COVID-19, *n* (*%*)*				
Student	1530 (25.1)	1126 (26.2)	304 (20.4)	100 (33.2)
Unemployed	667 (10.9)	424 (9.9)	173 (11.6)	70 (23.3)
Part-time employee	826 (13.6)	631 (10.4)	137 (2.2)	58 (1.0)
Full-time employee	2320 (38.1)	1582 (26.0)	688 (11.3)	50 (0.8)
Sick-leave	395 (6.5)	283 (4.6)	91 (1.5)	21 (0.3)
Retired	423 (6.9)	290 (6.7)	105 (7.1)	28 (9.3)
Marital status, *n* (*%*)				
Single	2632 (43.2)	1708 (39.7)	775 (52.0)	149 (49.5)
Living apart	736 (12.1)	532 (12.4)	155 (10.4)	49 (16.3)
Married/Partner	2443 (40.1)	1865 (43.3)	488 (32.8)	90 (29.9)
Divorced	255 (4.2)	175 (4.1)	68 (4.6)	12 (4.0)
Widow/widower	29 (0.5)	24 (0.6)	4 (0.3)	1 (0.3)
Birthplace, Sweden, *n* (*%*)	5687 (93.3)			

*Allowed for multiple response options.

**TABLE 5:** Self-reported life-time psychiatric disorder and suicidality*.

	Total (*n* = 6095)	Female (*n* = 4304)	Male (*n* = 1490)	Non-binary (*n* = 301)
	*n (%)*	*n (%)*	*n (%)*	*n (%)*
*Psychiatric disorders*				
Bipolar and related disorders	787 (12.9)	569 (13.2)	178 (12.0)	40 (13.3)
Major depressive disorder	4906 (80.5)	3544 (82.3)	1105 (74.2)	257 (85.4)
Anxiety disorders				
Specific Phobia	283 (4.6)	222 (5.2)	37 (2.5)	24 (8.0)
Social anxiety disorder	1160 (19.0)	838 (19.5)	245 (16.4)	77 (25.6)
Panic disorder	2567 (42.1)	1922 (44.7)	513 (34.4)	132 (43.9)
Agoraphobia	244 (4.0)	175 (4.1)	50 (3.4)	19 (6.3)
Generalized anxiety disorder	2795 (45.9)	2021 (47.0)	610 (40.9)	164 (54.5)
Somatic symptom and related disorder	427 (7.0)	313 (7.3)	103 (6.9)	11 (3.7)
Obsessive-compulsive and related disorder	572 (9.4)	433 (10.1)	108 (7.3)	31 (10.3)
Trauma- and stressor-related disorders	1252 (20.5)	980 (22.8)	186 (12.5)	86 (28.6)
Feeding and eating disorder	1071 (17.6)	912 (21.2)	80 (5.4)	79 (26.25)
Schizophrenia spectrum and other psychotic disorders	232 (3.8)	130 (3.0)	82 (5.5)	20 (6.6)
Substance-related and addictive disorders	605 (9.9)	304 (7.1)	269 (18.1)	32 (10.6)
Neurodevelopmental disorders	1458 (23.9)	968 (22.5)	364 (24.4)	126 (41.9)
*Suicidality*				
Non-suicidal self-harm	2726 (57.7)	2035 (60.5)	487 (43.5)	204 (85.0)
Suicidal ideation	3543 (75.0)	2481 (73.7)	854 (76.3)	208 (86.7)
Suicide attempt	1384 (29.3)	1005 (29.9)	295 (26.4)	84 (35.0)

*Allowed for multiple response options.

### Lifestyle behaviors and resilience in relation to COVID-19

**FIGURE 1:**
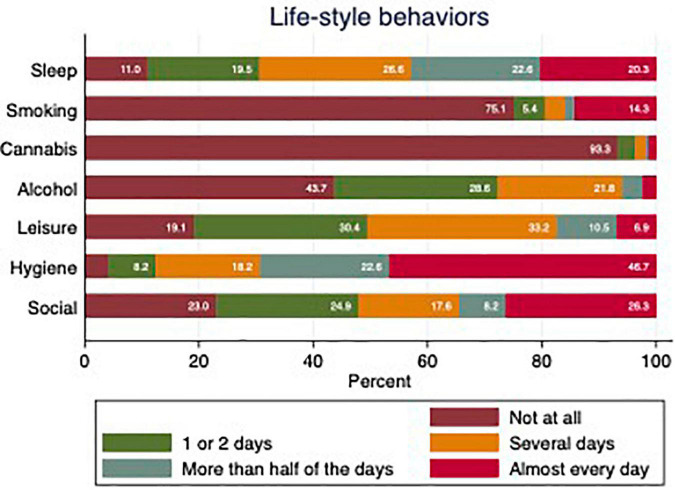
Life-style behaviors during the past 2 weeks during COVID-19. Sleep refers to “Got enough sleep.” Smoking refers to “Smoked cigarettes or vaped”. Cannabis refers to “Smoked cannabis.” Alcohol refers to “Drunk alcohol.” Leisure refers to “Taken part in leisure activities you enjoy.” Hygiene refers to “Maintained normal levels of hygiene.” Social refers to “Socialized with people inside your home.”

Half of the participants reported insufficient sleep on more than half of the days of the week, and a fifth of the participants did not at all take part in any leisure activities. These and other lifestyle behaviors of relevance, in relation to the pandemic are reported in Figure 1. On average, participants disagreed or were neutral to statements of being resilient. The pattern was found for the whole sample (*M* = 2.39, SD = 0.83), women (*M* = 2.35, SD = 0.81), men (*M* = 2.54, SD = 0.86), and non-binary (*M* = 2.18, SD = 0.81).

### Consequences of COVID-19 on work and social life

Regarding the present experience of feeling isolated, compared to before the COVID-19 pandemic, 4394 participants (72.1%) described feeling more isolated than before, 1513 participants described feeling the same as before (24.8%), and 188 participants described less (3.1%) feelings of isolation. As for contact with close family and friends, 4626 participants (75.9%) reported less interactions, 1253 participants (20.6%) the same as before, and 216 participants (3.5%) had more contact with close family and friends. Regarding work life, 730 participants (12.0%) reported becoming unemployed during the pandemic.

### Psychiatric symptoms and treatment during COVID-19

At baseline, the participants reported their psychiatric symptoms during the pandemic based on the different questionnaires administered. Psychiatric symptoms in our sample are in comparison to healthy control norm data (HCND) presented in Tables 2, 3.

Information on the established and/or change in mental health care contacts and treatments during COVID-19 are described in Table 6. Whereas 29% of the participants maintained their previously established mental health contact through the pandemic, a number of participants (22%) established new mental health contact. Furthermore, a majority of the participants reported an increase in their psychiatric medication use during the pandemic i.e., 20%, compared with 4% who reported a decrease in medication during the pandemic.

**TABLE 6:** Changes in health care contacts and treatment during the COVID-19 pandemic*.

	Total (*n* = 6095)	Female (*n* = 4304)	Male (*n* = 1490)	Non-binary (*n* = 301)
	*n (%)*	*n (%)*	*n (%)*	*n (%)*
Established mental health contact prior to COVID-19 and no change	1745 (28.6)	1255 (29.2)	394 (26.4)	96 (31.9)
No mental health contact before the COVID-19 pandemic and still do not feel that I need one	1259 (20.7)	846 (19.7)	359 (24.1)	54 (17.9)
No mental health contact before the COVID-19 pandemic but I have now initiated a contact	1336 (21.9)	983 (22.8)	293 (19.7)	60 (19.9)
Change from in-person meeting to telephone of video meeting	1372 (22.5)	1008 (23.4)	281 (18.9)	83 (27.6)
Decreased psychiatric symptoms and no need for regular contact with mental health care	403 (6.6)	265 (6.2)	116 (7.8)	22 (7.3)
Decrease in medication during COVID-19	248 (4.1)	161 (3.7)	74 (5.0)	13 (4.3)
Increase in medication during COVID-19	1246 (20.4)	937 (21.8)	246 (16.5)	63 (20.9)

*Allowed for multiple response options.

## Discussion

The current study presents an outline of a larger longitudinal investigation in Sweden that aims to explore the effects of the COVID-19 pandemic among individuals with self -reported psychiatric disorders. In addition, it has provided data on the participants’ demographics, social situation and a number of aspects related to their psychological well-being and general health during the 2 years of repeated outbreaks. Participants were recruited between July 2020 and June 2021 and the profile of participants in the study indicate that efforts in reaching out and recruiting the intended target group has been successful, and that it constitutes a diverse sample with regards to both background and psychiatric symptom profile. As such, the current study is an important contribution to the literature on the pandemic and its relationship to mental health problems, particularly as previous research typically only involved individuals from a non-psychiatric community population. To our knowledge this is the largest population-based survey study in Sweden, aiming to evaluate the impact of the pandemic in a risk group of individuals struggling with mental health symptoms.

During the time period from July to October 2020, the infection rate in Sweden were relatively low (37). Starting in October, the infection rate started rising and had two distinct peaks; one hitting at Christmas and New Year 2020 with around 7400 cases/7-day average. When the infection rate started rising during spring 2020, several regions in Sweden implemented new regulations, recommending people to abstain from physical contact with people outside one’s household, visiting public places as well as attending meetings, concerts, performances and sports training (and this also applied to older adolescents and young adults). Access to non-emergency medical care was made available via digital meetings. Vaccination started just before Christmas in 2020, beginning with the older population and those -at -risk (this did not include persons with serious mental illness). When infection rates peaked around Christmas in 2020, Sweden for the first time introduced the recommendation to wear a mask when traveling within public transport and in closed public spaces such as in offices. During early 2021 additional regulatory measures included restaurants, cafes and bars which were obliged to close at 8.30 pm, a visitors’ limit within shops, cafes and public areas and a total ban on all sports below elite level. These restrictions were in place during spring 2021 and it was not until May that the infection rate started to decline. At that time, the adult population had been offered the first dose of vaccine and by June vaccination was offered to adolescents from 16 years and over. During the whole period of data collection of the present study, Sweden had massive media coverage with live updates from the government several days a week. Together with the epidemiological situation and the accompanying regulatory measures, it is likely that these factors will have affected symptoms of anxiety and depression in general and also in this vulnerable population. Unlike other countries within the EU and globally, Sweden, however, did not have any periods of lock-downs. The pace of recruitment for our study during the 1 year, however, did not reflect the waves in the pandemic, but were more related to efforts of advertising via social media and this may be related to the voluntary nature of the study.

Almost all of the participants in the present study reported that they have had, or currently were experiencing, at least one life-time psychiatric disorder. In addition to self-reported diagnoses, the specific rates of the different psychiatric disorders (as assessed by validated clinical scales); major depressive disorder, panic disorder, and generalized anxiety disorder were highly prevalent in this population. For example, more than half of the participants had scores indicating moderate or severe anxiety, and moderately severe or severe depression. Although these numbers were much higher than prevalence rates among community-dwelling adults [e.g., (38, 39)], they are not unexpected, given that participants were recruited based on a history of psychological distress. In addition, comorbid conditions are common and might explain the current findings. In a synthesis of 76 studies by Ter Meulen et al. (40), three-quarters of all patients experience depression-anxiety comorbidity, making it a rule rather than exception. However, rates of substance-related and addictive disorders in the present study may be lower than what is usually found in a psychiatric population (41), possibly due to difficulties reaching individuals with a history of substance use, in studies of this nature.

Regarding lifetime diagnosed somatic problems, 38% reported having none of the most common chronic somatic disorders. Thirty-seven percent of the participants reported allergies, and 19% reported irritable bowel syndrome. A relatively high degree of participants (16%) also reported lung or breathing problems, which might be of interest to note in relation to the pandemic. The rate of diabetes reported in the study population (0.9 and 3.0% for type 1 and type 2, respectively) seems low, given that the Swedish Diabetic Association has reported that approximately 5% of the Swedish general population suffer from any type of diabetes (42).

A systematic review including 16 observational studies involving more than 19,000 patients across seven countries, demonstrated that individuals with mental health diagnoses had an increased risk of mortality when infected by COVID-19 (43). Concurrent diagnoses including impaired immune functioning which can co-occur with major mental health diagnoses and lack of access to adequate care could be some of the risk factors behind this association. Findings regarding physical health risk factors are therefore relevant in order to understand the degree to which individuals with mental health symptoms are vulnerable to COVID-19 infection. This sample constitutes individuals with both high levels of depressive and anxiety symptoms at baseline as well as a large proportion of concurrent somatic problems. This yields opportunities to further study and understand how this combination contributes to their symptom profile during the pandemic, such as if those with concurrent somatic problems show elevated anxiety and depressive levels over the course of the pandemic.

There were fluctuating patterns in the self-care behaviors during the pandemic. For example, on more than half the days of the week, only 43% reported adequate sleep, 17% reported having enjoyed leisure activities and 28% reported low levels of social interaction at home. A majority (69%), however, reported having maintained physical hygiene more than half of the days of the week. With regards to self-damaging behaviors, 16% of respondents reported smoking or vaping more than half of the days per week. This could be compared to the Swedish population at large, where 7% report being daily smokers and about 12–14% report daily or occasional smoking (44). Also, considering that tobacco smoking is two to three times higher among people living with mental illness (45–47), the result from the baseline assessment does not necessarily represent an increase. The finding that 5% of the respondents’ report cannabis use is also in line with epidemiological research (48). The 6- and 12-months follow-up assessments will be useful in gauging whether the COVID-19 pandemic and its related societal changes brought about any changes in either the self -care or self-damaging behaviors in this vulnerable population.

A substantial number of respondents (20%) reported an increase in psychiatric medication. A total of 29% had equal levels of care contacts related to mental health, and 22% reported having established new care contacts with psychiatric services during the study period. Taken together, these results imply a potential increase in care load within both psychiatric outpatient care as well as primary care settings for mental health problems during the pandemic. One way that mental health professionals have adjusted to the COVID-19 pandemic, is by increasing the amount of digital care contacts by offering video sessions via a secure online interface. Interestingly, 22% of the respondents in the present study reported canceling their mental health care contact due to a decrease in symptoms. This could possibly partly explain the fact that in Sweden several outpatient care facilities specifically within psychiatry reported a decrease in patient flow during the early phases of the COVID-19 pandemic (49). Whether this change was due to reduction in symptoms or due to the novelty or discomfort in accessing care digitally, is a question that needs further examination.

The participants in the current study constitute a vulnerable group in the sense that most reported at least one life-time psychiatric disorder, and that a majority reported high levels of psychiatric symptoms. This fits well with the purpose of the longitudinal investigation, which aims to understand how people with mental health problems in Sweden have fared during the COVID-19 pandemic. Accumulating data will be presented in a number of upcoming studies on several topics related to different symptoms more specifically and changes over time. Given the large set of demographics and number of instruments included, it will be possible to examine not only the response to the outbreak for different groups, but also compare the results to other similar initiatives around the world. For example, because different countries imposed different regulations and recommendations on dealing with the disease, investigating, and contrasting the effects of such measures on psychiatric symptoms might be feasible. Likewise, the relationship between psychiatric disorders and the risk of infection as well as mortality could be explored in greater detail by amassing and aggregating large datasets from different research groups (43).

However, the findings from the current study have already provided some insights into how individuals with psychiatric disorders in Sweden have responded to the COVID-19 pandemic with regard to healthcare contacts and medication. The overall picture seems to be complex, with some individuals having perceived an emerging need of support due to novel or increased psychiatric symptoms, while others canceled an established contact with a healthcare provider. This could be important to consider for clinicians and decision-makers when trying to administer mental health care following the COVID-19 pandemic, such as allocating increased resources to those sectors in a society experiencing a spike in referrals or admissions. It might also be warranted to explore what type of care is needed (e.g., more resources allocated to digital health care) and in addition what developments are needed (e.g., taking into account the needs of different age groups, digital literacy also for personnel working within psychiatric services) to make in the existing formats of mental health care to better address the needs. Investment in preventive strategies and conditions to maintain and promote mental health will be also key focus areas going forward. Meanwhile, researchers should also explore why some individuals with a history of mental health problems actually improved in their symptoms during the same period and required less healthcare contact. Did this subgroup cope with the outbreak and changes in society differently, or will they experience a delayed increase in symptoms? Following these participants over time using quantitative measures would shed some light on long-term outcomes, while qualitative interviews may help to gain a deeper understanding of how sudden and longer lasting changes in society may be differentially experienced by specific groups.

Meanwhile, how these participants’ psychological well-being and general health might change over time will be possible to discern given the longitudinal design of the project. The questionnaire also involves aspects such as resilience, self-care, healthcare contacts and medication. This creates a unique opportunity to understand how the COVID-19 pandemic has affected a vulnerable population with psychiatric symptoms, not only following the outbreaks but also in the long-term. As such, it has the potential of making a significant contribution to the literature, which might help researchers, clinicians, and decision-makers grasp the consequences of the COVID-19 pandemic on one of society’s most vulnerable groups.

## Limitations

This is a large community-based study where participants were recruited with the specific aim of reaching a heterogeneous sample across the country. One strength is the large sample size. Also, we seem to have captured the intended target population given that over 90% of the participants self-reported a certain current or life-time psychiatric disorder. As life-time psychiatric disorder was chosen from a list of disorders, there could also be underreporting as participants might not have known the exact disorder they had or not recognized the categories. Nevertheless, self-reported diagnoses have limitations in the sense that they are not clinical diagnosis and in the present study we describe the mental health symptoms reported by this specific population. Our ambition was to recruit participants from diverse ethnic backgrounds across Sweden using targeted ads in social media, however, it was difficult to obtain an ethnically diverse sample. This is in line with public reports in Sweden demonstrating difficulties reaching out with information about COVID-19 vaccination in marginalized neighborhoods. The generalizability of the results could be impacted by attrition; in the case of questionnaires regarding current depressive and anxiety symptoms, 77–82% provided data, while the number for and alcohol and substance use symptoms was 74%. The participants in the current study were relatively young (*m* = 35), which could be compared to the average age in Sweden in 2021 being 42 years. One potential reason for the younger age, might be that a significant portion of study advertising took place via social media platforms. Although efforts were made to systematically target and recruit different age groups during the ongoing pandemic, it seems that these efforts were insufficient. Thus, the results from the current study should be interpreted with this limitation in mind. Another limitation concerns the lack of pre-pandemic data and the lack of a comparison control group sampled from the general population. Nevertheless, investigating changes over time during the different waves of pandemic, which is the main objective of the longitudinal study, is important in order to better understand what interventions may be beneficial for individuals with psychiatric conditions in this type of situation.

## Conclusion

Baseline data from this longitudinal cohort in Sweden demonstrate that individuals with pre-pandemic psychiatric symptoms represent a vulnerable population with regard to their responses to the COVID-19 pandemic. Levels of anxiety and depressive symptoms were high, and in this group, and one-fifth of the participants reported an increase in usage of psychiatric medication during the pandemic. A substantial number of participants also reported co-existing somatic diagnoses and symptoms. The follow up data will further indicate how health, well-being and access to care in this population developed during the continuation of the pandemic. Our data provides a foundation for understanding what are the important aspects to consider in psychiatric vulnerable populations for future outbreaks or other country specific or global crisis.

## Data availability statement

The original contributions presented in this study are included in the article/supplementary material, further inquiries can be directed to the corresponding author.

## Ethics statement

The studies involving human participants were reviewed and approved by Swedish Ethical Review Authority (Dnr: 2020-02798). The participants provided their written informed consent to participate in this study.

## Author contributions

OO and SEA set up the REDCap study platform and structure for all the questionnaires and follow-up modules within the system and managed the administration of the entire study via REDCap. OO, SJ, SEA, and KM contributed equally to the data management, analysis, and results. AR, KS, and NJ-L contributed equally in drafting of the manuscript. All authors have been part of the study from the start, have read and contributed to the preparation of the final manuscript.

## Conflict of interest

The authors declare that the research was conducted in the absence of any commercial or financial relationships that could be construed as a potential conflict of interest.

## Publisher’s note

All claims expressed in this article are solely those of the authors and do not necessarily represent those of their affiliated organizations, or those of the publisher, the editors and the reviewers. Any product that may be evaluated in this article, or claim that may be made by its manufacturer, is not guaranteed or endorsed by the publisher.

## Footnotes

1
www.psykiskohalsacovid19.se
